# C–H oxidation in fluorenyl benzoates does not proceed through a stepwise pathway: revisiting asynchronous proton-coupled electron transfer†

**DOI:** 10.1039/d1sc03344a

**Published:** 2021-09-10

**Authors:** Scott C. Coste, Anna C. Brezny, Brian Koronkiewicz, James M. Mayer

**Affiliations:** Department of Chemistry, Yale University New Haven CT 06520-8107 USA james.mayer@yale.edu; Department of Chemistry, Skidmore College Saratoga Springs New York 12866 USA

## Abstract

2-Fluorenyl benzoates were recently shown to undergo C–H bond oxidation through intramolecular proton transfer coupled with electron transfer to an external oxidant. Kinetic analysis revealed unusual rate-driving force relationships. Our analysis indicated a mechanism of multi-site concerted proton–electron transfer (MS-CPET) for all of these reactions. More recently, an alternative interpretation of the kinetic data was proposed to explain the unusual rate-driving force relationships, invoking a crossover from CPET to a stepwise mechanism with an initial intramolecular proton transfer (PT) (Costentin, Savéant, *Chem. Sci.*, 2020, **11**, 1006). Here, we show that this proposed alternative pathway is untenable based on prior and new experimental assessments of the intramolecular PT equilibrium constant and rates. Measurement of the fluorenyl 9-C–H p*K*_a_, H/D exchange experiments, and kinetic modelling with COPASI eliminate the possibility of a stepwise mechanism for C–H oxidation in the fluorenyl benzoate series. Implications for asynchronous (imbalanced) MS-CPET mechanisms are discussed with respect to classical Marcus theory and the quantum-mechanical treatment of concerted proton–electron transfer.

## Abstract 2

2-Fluorenyl benzoates were recently shown to undergo C–H bond oxidation through intramolecular proton transfer coupled with electron transfer to an external oxidant.

## Graphical abstract

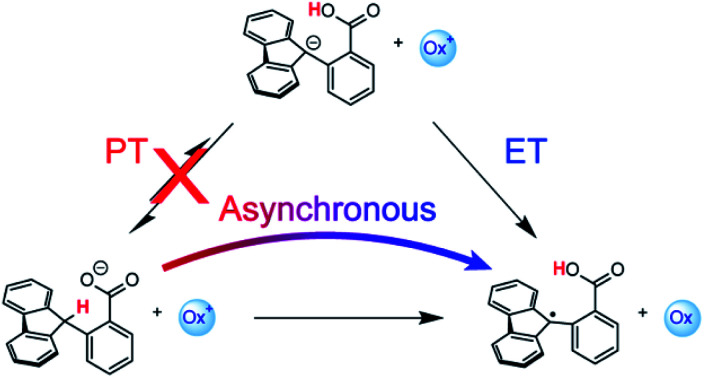

## Introduction

Rate-driving force relationships of proton-coupled electron transfer (PCET) reactions provide essential mechanistic and kinetic insight into organic transformations, biochemical reactions, and industrial processes.^1–3^ The simplest connection between rate and equilibrium constants is the Brønsted catalysis ‘law,’ linearly relating the logarithms of these two quantities with a slope *α* (eqn (1)). Originally developed for acid-catalysed reactions, the Brønsted equation is now recognized as a linear free energy relationship (LFER), usually using the Eyring equation to convert ln(*k*) to the free energy barrier Δ*G*^‡^. Marcus theory predicts a quadratic relationship between Δ*G*^‡^ and Δ*G*°, with a slope that varies with the ratio of the driving force to the intrinsic barrier *λ*.^4^ At low driving forces, Δ*G*° ≪ 2*λ*, *α* is predicted to be close to 0.5 (at constant *λ*). When applied to a single elementary kinetic step, in the context of the Hammond postulate, *α* often qualitatively describes the nature (or progression) of the transition state relative to reactant and product structures.

1 *α* = ∂log(*k*)/∂log(*K*_eq_) = ∂Δ*G*^‡^/∂Δ*G*°

2 *α*(Marcus) = 0.5 + Δ*G*°/2*λ*

The Marcus-predicted dependence of *α* on Δ*G*° has been observed for a number of concerted proton–electron transfer (CPET) reactions. For instance, *α* values close to 0.5 have been reported for reactions ranging from hydrogen atom transfer (HAT)^5^ to multi-site CPET (MS-CPET).^6^ On the other hand, several systems have shown deviations from this behaviour.^7^ For example, Qiu and Knowles' study of photochemical MS-CPET reductions of ketones with Δ*G*°′ = +10 to −8.5 kcal mol^−1^ found *α* = 0.17, surprising for the small value and excellent linearity over this 0.8 eV range of driving forces.^7*d*^ Explaining the reasons for such discrepancies within the context of classical Marcus theory remains an ongoing discussion.

**Scheme 1:**
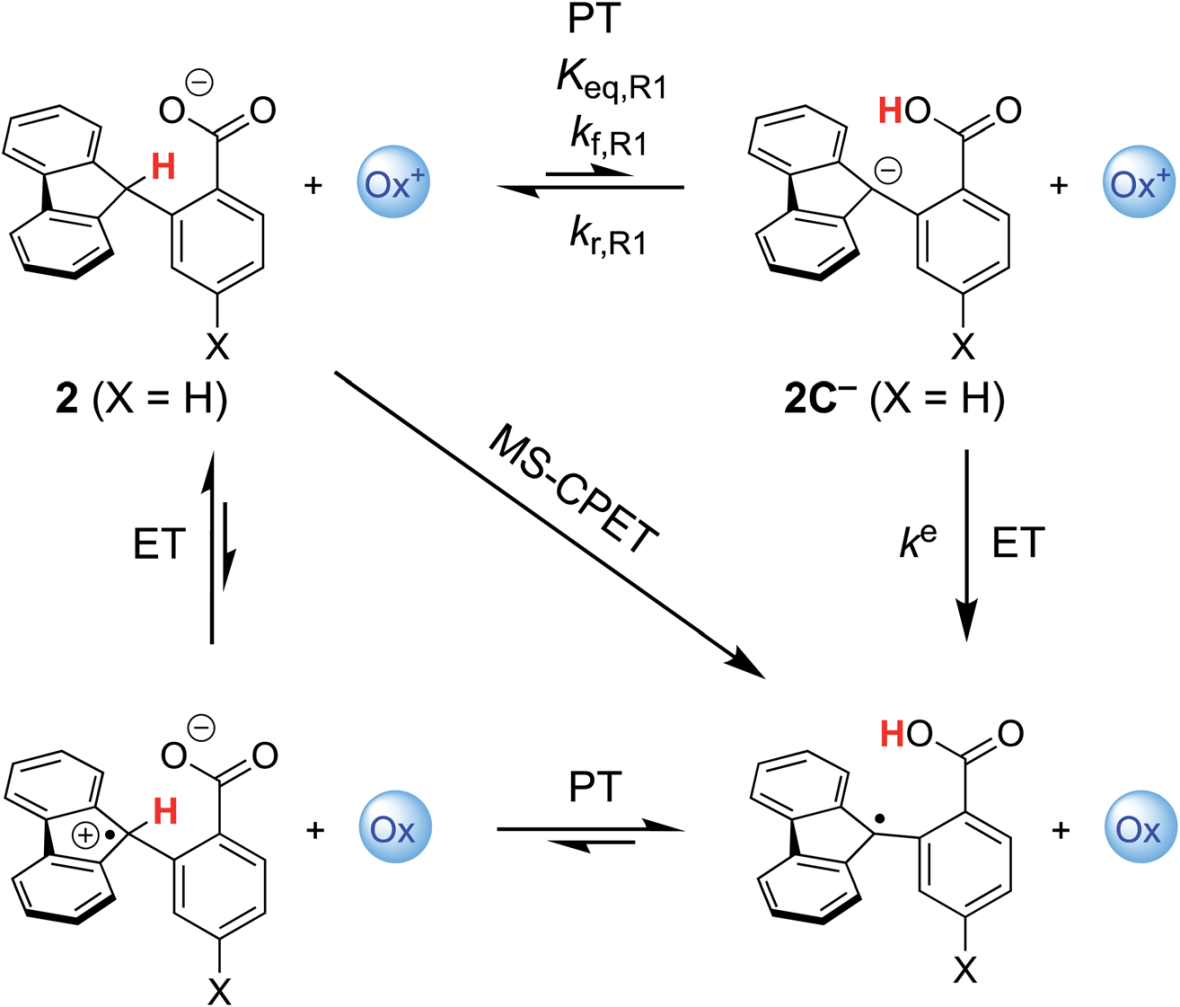
Square scheme depicting paths for the homolytic C–H oxidation in 2-fluorenyl benzoates with an external oxidant. The concerted MS-CPET path is on the diagonal, with the two stepwise pathways around the square: PT followed by ET or ET followed by PT. Relevant thermochemical and kinetic parameters are depicted for discussion below. Adapted from ref. 13.

Our group previously reported homolytic C–H oxidation through MS-CPET in a fluorenyl benzoate series (Scheme 1) using external 1e^−^ oxidants.^8^ Similar to the above example, we observed *α* to be constant over the entire range of oxidant driving forces (spanning 26.5 kcal mol^−1^ or 1.15 eV) and shallow (*α* = 0.23), deviating from that expected of classical Marcus theory. Theoretical analysis of the MS-CPET reaction, where both the proton and electron are treated quantum mechanically, predicted a low *α* value of 0.37.^9^ This is good agreement given the complexity of the system. Application of this nonadiabatic PCET theory attributed the low value to the involvement of excited vibronic states in the homolytic C–H bond cleavage.

More recently, we reported that the dependence of *k*_CPET_ on 
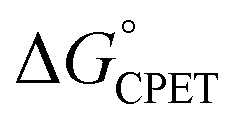
 in this system was quite different when the driving force was varied through changing the substituent X in the *para* position of the benzoate (Scheme 1).^10^ Changing X through the series CF_3_, H, OMe, NH_2_ was suggested to primarily increase the basicity of the carboxylate. Comparing these four substrates with a given oxidant showed a larger *α*, in the range 0.48–0.61 for five of the seven oxidants studied (with outliers at 0.36 and 0.99, experimental uncertainties all ±0.1). Thus, in contrast to a Marcus analysis, there was not a 1 : 1 correspondence between *k*_CPET_ and 
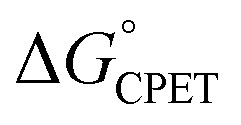
. A particular 
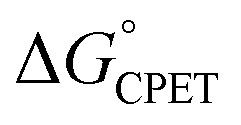
 gave different rate constants depending on whether it was obtained with a weaker oxidant and a more basic carboxylate, *vs.* a stronger oxidant and less basic carboxylate. This behaviour was suggested to be an experimental marker for asynchronous CPET, building on prior indications that sets of PCET reactions appear to respond differently to changes in p*K*_a_ and *E*°.^11,12^

Costentin and Savéant (CS) responded to our report with an alternative interpretation of the data, that the apparently unusual behaviour was simply the result of a change in mechanism.^13^ They suggested that the reactions at high driving force occurred by CPET but those at low driving force occurred by the stepwise path of pre-equilibrium PT followed by rate limiting ET. Their proposal was likely motivated by their belief that CPET cannot occur in an asynchronous manner, and was supported by our report of an inaccurate DFT-calculated value of the free energy of proton transfer (+6.5 kcal mol^−1^).^10^ We had previously reported a much larger calculated 
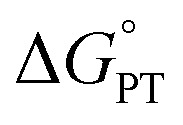
 of +16.5 kcal mol^−1^,^8^ which is shown here to be much closer to experiment. Both of these values were computed for qualitative insights and neither was used (or intended to be used) in mechanistic analysis,^14^ so CS's choice of the lower value was unfortunate.

Our first report on this system ruled out the stepwise PT-ET mechanism largely based on the experimental lack of H/D exchange overnight between deuterated **2** (**2CD**) and MeOH.^8,15^ We assumed that the carboxylic acid formed by initial PT would rapidly exchange with the excess MeOH, and therefore the lack of exchange of the fluorenyl CD implied a very slow rate of initial PT (*k*_f,_**R1** in Scheme 1). The slowest of the oxidations reported in this system had a half-life of about six seconds, and the overall rate of the PT-ET pathway could not be faster than *k*_f,_**R1**, so we argued that the lack of any H/D exchange overnight ruled out initial PT. Strangely, this pathway was not considered by CS, who apparently mistakenly assumed that the only path to the deuterated product was *via* direct protonation of the carbanion by methanol (page 1 of the ESI† from ref. 13). We have since shown that H/D exchange between protio substrates and MeOD occurs under photoredox conditions, implying exchange between the carboxylic acid proton and methanol as part of the mechanism.^16^ In addition, analyses of these reactions of substituted compounds by nonadiabatic PCET theory explained the large *α* values as resulting from changes in ground state structures.^17^ For these various reasons, we were confident that CS's alternative interpretation of our data was not correct.

Nonetheless, understanding the origins of the rate-driving force relationships in the fluorenyl benzoate system is critical for contextualizing its application towards homolytic cleavage of C–H bonds in other systems.^18^ So, the validity of the alternative model deserves attention. Other PCET systems with unusual rate-driving force relationships, such as tungsten hydrides, have been shown to exhibit mechanism crossover from CPET to stepwise processes,^19^ which was likely part of CS's inspiration to propose the alternative mechanism. Such crossovers depend critically on the free energies for the competing pathways,^19*c*^ so we thought it best to experimentally assess the 
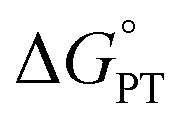
 for the proposed alternate mechanism. Herein, we report experimentally measured p*K*_a_s, kinetic measurements of H/D exchange, and kinetic modelling. These results show that the *K*_eq,_**R1** for initial proton transfer is too low to account for the measured C–H oxidation rates at low driving force, ruling out the PT-ET mechanism proposed in ref. 13. Having reaffirmed the unusual Brønsted *α* values in this system, we finish with a discussion of their origins, in the broader context of asynchronous or imbalanced concerted PCET reactions where the PT and ET components do not contribute equally to the observed rate constants.

## Results and discussion

### (I) Estimating the *K*_eq_ for pre-equilibrium PT

Elucidating the involvement of pre-equilibrium PT in the oxidation of the fluorenyl C–H bond in Scheme 1 first requires knowledge of its thermochemistry. CS proposed a stepwise path consisting of rapid, intramolecular pre-equilibrium PT (*K*_eq,_**R1**) followed by rate-determining ET (*k*^e^), giving eqn (3) as the expression for the measured bimolecular oxidation rate constant, *k*_MS-CPET_. The feasibility of this model therefore depends on the value of the equilibrium constant. We term the initial equilibrium constant *K*_eq,_**R1** because this is reaction (1) in the kinetic model described in Section III below.

3 *k*_MS-CPET_ = *K*_eq,_**R1***k*^e^

**Scheme 2:**
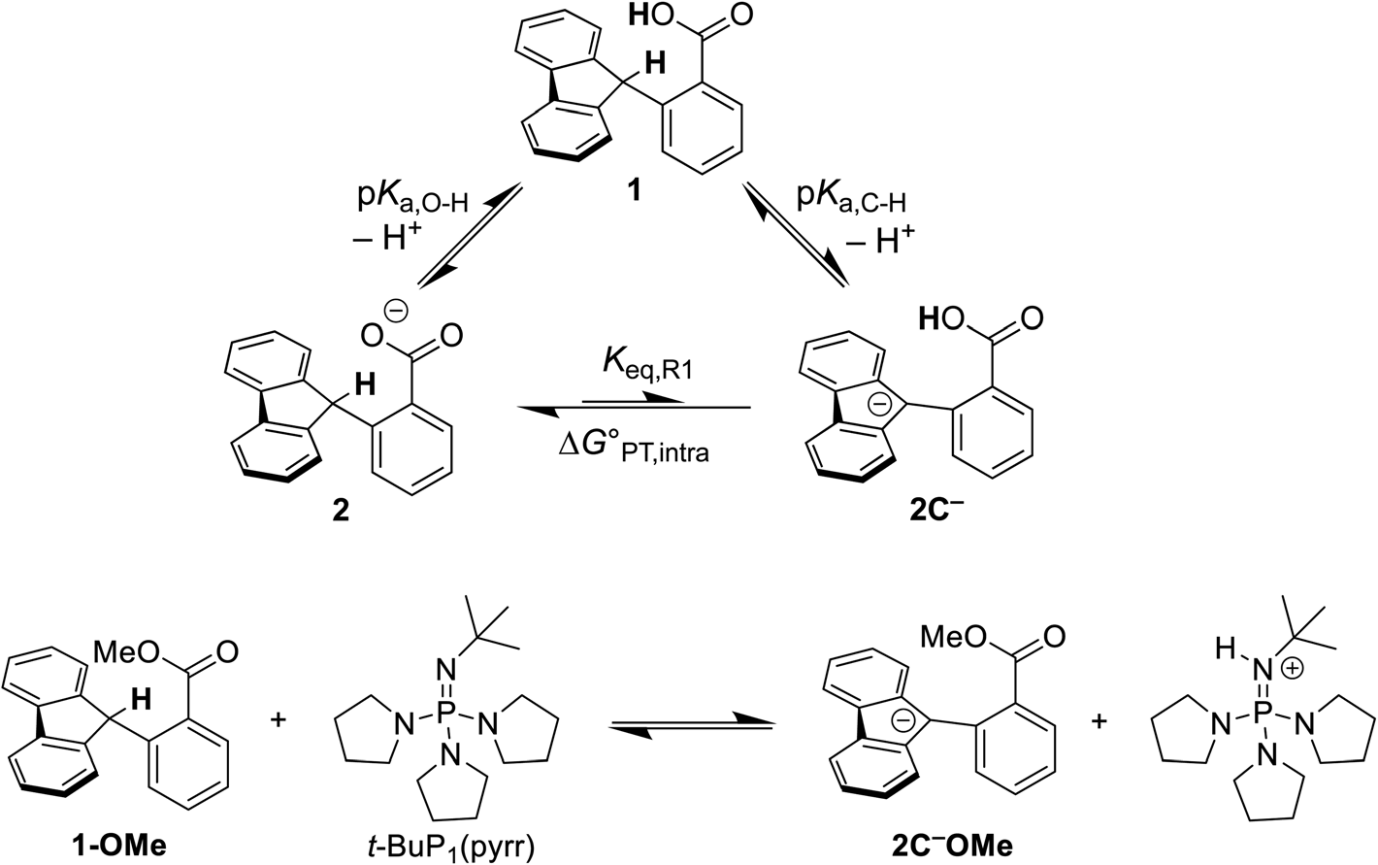
(Top) Thermochemical cycle to estimate the 
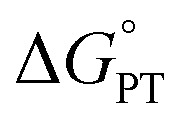
 for intramolecular proton transfer. (Bottom) Equilibrium formed between **1-OMe** and *t*-BuP_1_(pyrr).

The intramolecular equilibrium constant *K*_eq,_**R1** is given by the difference in p*K*_a_s of the carboxylic acid and fluorenyl protons (Scheme 2, top). The benzoic acid p*K*_a_ in **1** was previously measured to be 21.3 in MeCN.^10^ The acidity of the fluorenyl proton in **1** cannot be measured directly because the acid deprotonates first, so we used the methyl ester derivative **1-OMe**. The fluorenyl C–H bond p*K*_a_ in this ester should be similar to that of the carboxylic acid **1**, because the COOH and COOMe groups are unchanged in the deprotonation reaction (the carbanion–carboxylic acid **2C−** likely has a substantial C^−^⋯HO interaction, but this is in competition with the OH group hydrogen-bonding to the solvent^20^ and therefore does not provide significant additional stabilization; see ESI Section 3.4†). The p*K*_a_ in the ester was determined by a spectrophotometric titration, using the strong optical absorbance of the fluorenyl anion to determine its concentration (see ESI†).^21^ Titration of **1-OMe** with the strong phosphazene base, *tert*-butylimino-tri(pyrrolidino)phosphorane (*t*-BuP_1_(pyrr)), forms an equilibrium with **2C−OMe** (Scheme 2, bottom). This strong base was chosen due to the low acidity of the carbon acid, and because its steric bulk minimizes ion pairing and hydrogen bonding. Using the standard mass balance equations, the optical data showed the equilibrium constant to be 1.5 ± 0.4 × 10^−3^ (ESI†). Given the p*K*_a_ of *t*-BuP_1_(pyrr) (28.4 in MeCN),^22^ this establishes the p*K*_a_ of the fluorenyl 9-C–H bond in **1-OMe** to be 31.2 ± 0.1. This should be a very close estimate of the fluorenyl p*K*_a_ in **1**.

Combining the p*K*_a_s of the fluorenyl and benzoic acid protons gives *K*_eq,_**R1** of 1.3 ± 0.3 × 10^−10^ for the pre-equilibrium intramolecular PT in **2**. The *K*_eq,_**R1** is consistent with the initial DFT calculations used in the computational screen that led to the choice of **2** as a candidate for an MS-CPET mechanism.^8^ However, this value for *K*_eq,_**R1** is 10^5^ smaller than the value chosen by CS, from our computation of the internal reaction coordinate (IRC) in the subsequent paper.^10,13^ If the PT-ET mechanism suggested by CS were to hold, this measured value of *K*_eq,_**R1** requires that *k*^e^ be 10^2^ faster than computed by CS. The slowest observed oxidation rate constant for **1**, 12 M^−1^ s^−1^ by FeCp*_2_^+^, would require *k*^e^ to be 9 × 10^10^ M^−1^ s^−1^. This value is three hundred times larger than that maximum possible *k*^e^ in the CS analysis, given as *Z*^e^_2_ = 3 × 10^8^ M^−1^ s^−1^,^13^ and larger than the diffusion limit in MeCN (∼2 × 10^10^ M^−1^ s^−1^).^23^ Thus, this new experimental estimate of the fluorenyl proton acidity shows that the mechanism of pre-equilibrium PT followed by rate-limiting ET suggested by CS is not viable.

### (II) H/D exchange as a probe of initial PT

**Scheme 3:**
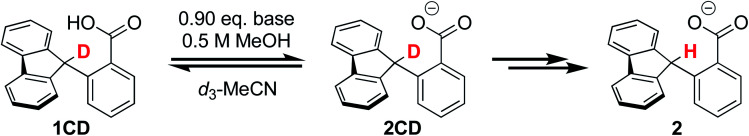
H/D exchange experiments where base is either TBAOH or TBAOBz.

**Fig. 1:**
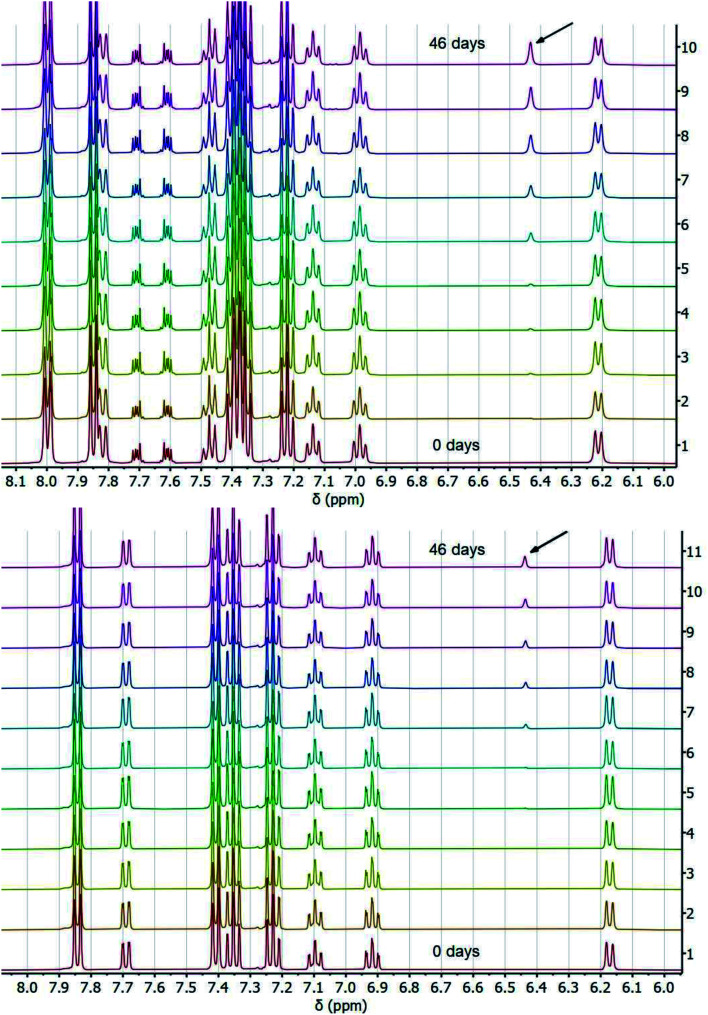
Stacked ^1^H NMR spectra of H/D exchange experiments with TBAOBz (top) or TBAOH (bottom) as a base over the course of 46 days at room temperature. Resonance at ∼6.43 ppm (indicated by arrow) shows growth of fluorenyl 9H proton.

**Fig. 2:**
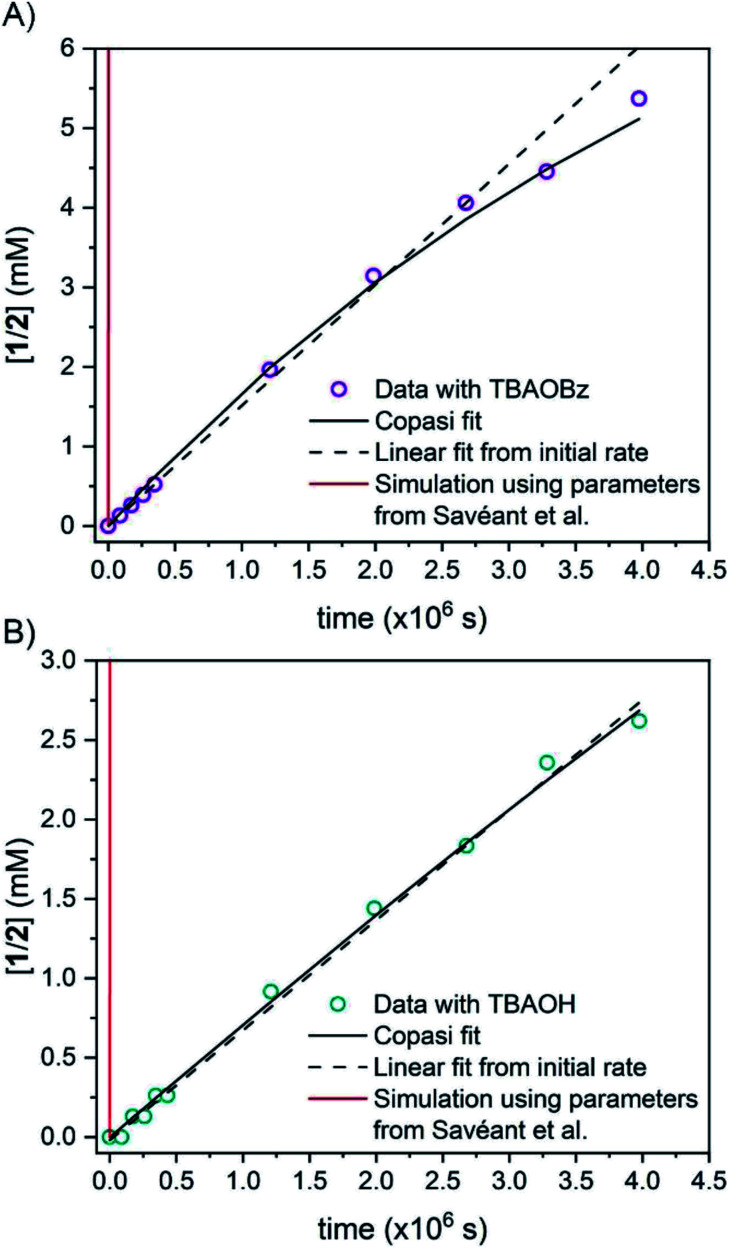
Concentration of protio 2-fluorenyl benzoate (**1**/**2**) over time at room temperature with (A) TBAOBz or (B) TBAOH as a base (open circles) as measured by integration of ^1^H NMR spectra. Horizontal scale is more than six weeks. Solid black lines are fits from COPASI parameter estimation function; dashed lines are the initial linear fit to the first 5 data points. The solid red lines are the predicted exchange using values from the CS analysis,^13^*K*_eq,_**R1** = 1.5 × 10^−5^, *k*_f,_**R1** = 4.3 × 10^4^ s^−1^ and *k*_r,_**R1** = 2.8 × 10^9^ s^−1^; these clearly do not fit the observations (see below).

To corroborate our measurement of *K*_eq,_**R1**, and to investigate the rate of intramolecular PT, we examined the kinetics of H/D exchange starting with compound **1** deuterated at the fluorenyl 9-position (**1CD**, Scheme 3). The kinetics of proton exchange were measured under conditions similar to those used for the C–H oxidation reactions in ref. 8 and 10, just without the addition of oxidant: 13.1 mM **1CD** with 0.9 equivalents of base added and 0.5 M MeOH in dried *d*_3_-MeCN at room temperature. The H/D exchange rate was measured by monitoring the growth of the integral of the 9-H proton in **2** by ^1^H NMR spectroscopy over six weeks (=3.6 × 10^6^ s) as shown in Fig. 1. The initial experiment used tetra-*n*-butyl ammonium hydroxide (TBAOH) as the base because that was used in the C–H oxidation reactions, and to roughly follow an experiment in ref. 8. The growth of protio-compound **2** was very slow, with only 10% H incorporation at the fluorenyl 9 position after 3 weeks (Fig. 2B, open circles).

H/D exchange was then examined using the weaker base TBA-benzoate (TBAOBz). This was stimulated by the comment in ref. 13 that methanol was likely too weak of an acid to protonate the fluorenyl anion. This supposition is correct, as the methyl ester **1-OMe** was deprotonated by NaOMe in MeCN to form the fluorenyl anion (ESI†). However, this was not the H/D exchange pathway that we proposed in our original paper.^8^ Using benzoate as a base has the advantage that benzoic acid is a much stronger acid than methanol.

**1CD** was reacted with 0.90 equivalents of TBAOBz to give an equilibrium mixture with close to a 1 : 1 ratio of **1CD** to **2CD** (*K*_BzO_ = 2.1) because the p*K*_a_ of **1** is very similar to that of benzoic acid. In this reaction, the total concentration of benzoic acids ([**1CD**] + [**1**] + [BzOH]) is constant over time, at the starting concentration of **1CD**, 13.1 mM, as the overall reaction does not consume acid. Exchange was monitored by the ^1^H NMR integral of the fluorenyl proton in the **1**/**2** mixture, which are in rapid equilibrium with each other and the various hetero- and homoconjugates present in solution (see **R4** in Scheme 5 below^24,25^). Under these conditions with the benzoate base, the growth of the 9-fluorenyl proton in **1**/**2** was slightly faster than with the corresponding hydroxide: 10% H incorporation was reached after 2 weeks (Fig. 2A, open circles). The initial rate of exchange under these conditions was 1.5 ± 0.1 × 10^−9^ M s^−1^ giving a pseudo-first order rate constant of *k*_ex_ = 1.8 × 10^−7^ s^−1^. Overall, H/D exchange is very slow regardless of the acid p*K*_a_ present in solution.

To analyse this rate of exchange, we have experimentally estimated the rate of protonation of the fluorenyl anion by benzoic acid. A 0.2 mM solution of the methyl ester fluorenide **2C−OMe** in MeCN (generated from **1OMe** and NaOMe) was mixed in a stopped-flow instrument with 0.2 mM benzoic acid in MeCN (see ESI†). Only ∼1% of the red fluorenyl anion was observed in the very first spectrum, at 5 ms during the mixing time of the instrument. This experiment provides a rough estimate of ∼1 × 10^6^ M^−1^ s^−1^ for the protonation rate constant, which is a good approximation for the rate of protonation of **2C−** by benzoic acid.

**Scheme 4:**
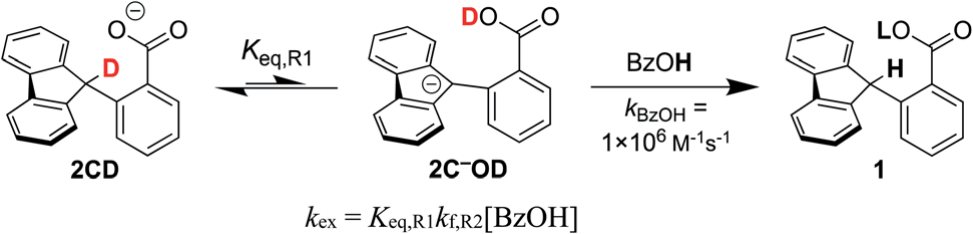
CS suggested mechanism for H/D exchange with benzoic acid as the protonation source.

This rate constant can be used to test the mechanism for H/D exchange in **2CD** that was proposed by CS:^13^ initial intramolecular proton transfer followed by protonation by benzoic acid (Scheme 4, L = H or D). The first-order rate constant for exchange would then be as shown in eqn (4), where all of the terms are known (ignoring for the moment that initial deprotonation of **1CD** by TBAOBz does not proceed to completion). The derived first approximation to *k*_ex_ is 2 × 10^−6^ s^−1^, in good agreement with the experimental value of 1.8 × 10^−7^ s^−1^. While a more quantitative analysis of these kinetics is given in the next section, this result supports the *K*_eq,_**R1** value determined using Scheme 2 above. Use of the *K*_eq_ chosen by CS would predict a 10^5^ faster rate constant for H/D exchange, as shown in Fig. 2 and discussed below.

4 *k*_ex_ = *K*_eq,_**R1***k*_f,_**R2**[BzOH]

### (III) Kinetic modelling of H/D exchange

To analyse the H/D exchange rates in more detail, we have created kinetic models accounting for various H/D exchange pathways in the presence of either base, TBAOBz or TBAOH. The model was fit globally to both data sets using COPASI.^26^ The goals of this effort were to put the rough estimate in Section II on a firmer footing, to determine whether the experimental estimate of *K*_eq,_**R1** is consistent with the H/D exchange experiments using a much more complete model, to test whether this value could be consistent with the PT-ET mechanism suggested by CS, and to inform on the mechanism of the H/D exchange.

**Scheme 5:**
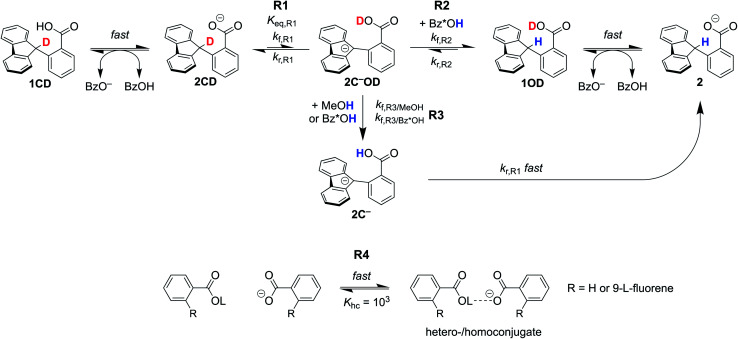
Kinetic scheme for H/D exchange modelled in COPASI. ^*a*^L = H or D. Bz*OH = **1**, **1CD**, or benzoic acid. All of the benzoic acids and benzoates under hetero/homoconjugation (**R4**). See ESI† for a complete discussion.

The kinetic models are shown in Scheme 5. The only difference between the TBAOBz and TBAOH scenarios was the inclusion of an equilibrium between **1**/**2CD** and benzoic acid prior to reaction (1) (**R1**) in the former case. For clarity, we note that compound numbers **1***vs.***2** are meant to distinguish between the neutral and anionic forms of the fluorenyl benzoate, respectively. The following designation **CD***vs.***OD** are meant to distinguish between isotopomers where the deuteron resides on the carbon or oxygen atom, respectively. **2C−** specifies that the compound is a carbanion rather than a carboxylate. Fast equilibration between **1CD** and the benzoate base forms an equilibrium amount of **2CD**. Intramolecular PT of **2CD** to the carbanion **2C−OD** (tautomerization, reaction **R1**) has the rate constants *k*_f,_**R1** and *k*_r,_**R1** and the *K*_eq,_**R1** measured in Section I above. Subsequently, two pathways can yield protonation at the fluorenyl 9-position. The first pathway involves direct, bimolecular protonation of the carbanion by benzoic acid, **1**, or **1CD** (reaction **R2**). These three benzoic acids, together denoted as Bz*OH in Scheme 5, are assumed to have the same rate constant *k*_f,_**R2**. The large excess of MeOH in solution (0.5 M) maintains all of the benzoic acids (Bz*OH) in the protio form throughout the weeks-timescale of the reaction. The second pathway involves exchange of the benzoic acid proton in **2C−OD** with Bz*OH or MeOH (**R3**), which is followed by rapid intramolecular PT (the reverse of **R1**) to give **2**. This latter path, **R3** then **R1**_r_, was our original proposal^8^ and was necessary to explain the photo-redox H/D exchange of **2CD** and other benzylic-carboxylate compounds with MeOD.^16^ All of the benzoate and benzoic acid species are assumed to undergo rapid hetero-/homoconjugation (**R4**) with an equilibrium constant of 10^3^ M^−1^.^24,25^ The presence of equilibria **R4** affect the concentration of free acid present, as discussed below.

The H/D exchange data in Fig. 2 were fit by COPASI using this kinetic model, to estimate the equilibrium constant for **R1** and the rate constant for **R2**, *K*_eq,_**R1** and *k*_f,_**R2**. Other rate constants were estimated, as described in ESI Section 5.1;† all relevant parameters are given in Table 1. In particular, our initial estimate of the rate of H/D exchange between benzoic acid protons, *k*_f,_**R3**_/Bz*OH_, was taken as diffusion limited, 10^10.2^ M^−1^ s^−1^, based on indications from other PT reactions.^27^ With this model, the best fit for *k*_f,_**R2** was 2.4 ± 0.1 × 10^6^ M^−1^ s^−1^, very close to what we estimate based on stopped-flow measurements. *K*_eq,_**R1** was not well fit in the model, estimated to be 1.0 ± 2000 × 10^−15^. This is much lower than (but within the uncertainty of) what we predicted from p*K*_a_ measurements in Section I, 1.3 ± 0.3 × 10^−10^. Analysis of the simulations shows that the refined *K*_eq,_**R1** value is tightly coupled to the rate constant chosen for the benzoic acid H/D exchange, *k*_f,_**R3**_/Bz*OH_. If *k*_f,_**R3** is lowered to ∼10^6^ M^−1^ s^−1^, the optimized *K*_eq,_**R1** is brought into agreement with the experimental value from Section I.

**Table 1:** Fitted and fixed parameters for kinetic model. Values fit using COPASI have reported uncertainties^a^

Parameter	Value fixed in the model	Value estimated by COPASI
*K*_eq,_ **R1**		(1.0 ± 2000) × 10^−15^
*k*_f,_ **R2**		(4.4 ± 0.1) × 10^6^ M^−1^ s^−1^
*k*_r,_ **R2**		(5.5 ± 0.1) × 10^−4^ M^−1^ s^−1^^b^
*k*_f,_ **R3**_/MeOH_	3.2 × 10^4^ M^−1^ s^−1^^c^	
*k*_f,_ **R3**_/Bz*OH_	10^10.2^ M^−1^ s^−1^^d^	

a See ESI Section 5 for complete details.

b *k*_r,_ **R2** set equal to *k*_f,_**R2**/10^9.9^, where 10^9.9^ = *K*_eq,_**R2**, estimated from the p*K*_a_ measurements in Section I.

c Obtained from ^1^H NMR linewidth measurements; see ESI Section 5.2.

d Taken to be at the diffusion limit.^27^

Simulations of the fitted parameters revealed that the rate of H/D exchange is affected only by *K*_eq,_**R1** and not its component rate constants *k*_f,_**R1** and *k*_r,_**R1**. This is because the rate of back-tautomerization (*k*_r,_**R1**) is much faster than any of the other reactions of **2C−OD** under the reaction conditions. This supports the assumption above that **R1** is a fast pre-equilibrium. We note that CS estimated a pre-exponential factor of 6 × 10^7^ s^−1^ for *k*_f,_**R1** and *k*_r,_**R1**;^13,28^ using this value with *K*_eq,_**R1** gives an upper limit for *k*_f,_**R1** of 5.0 × 10^−3^ s^−1^ which is too slow to account for the observed overall rates of C–H bond oxidation. To illustrate the effect of the larger *K*_eq,_**R1** value chosen by CS, each panel of Fig. 2 shows a simulation of the data using that larger *K*_eq,_**R1** value, as a red line (ESI Section 5.3†). In this scenario, H/D exchange would be complete in less than 1 minute as opposed to months. Therefore, the larger *K*_eq,_**R1** value in ref. 13 is necessary to obtain fast enough rates for the proposed PT-ET pathway by CS, but it clearly cannot account for the very slow H/D exchange rates here. The H/D exchange data require a significantly lower *K*_eq,_**R1** value to account for the observed rates, in agreement with our assessment in Sections I and II. This further demonstrates the mechanism crossover scenario proposed by CS is not possible.

The kinetic simulations show why the rate of H/D exchange using TBAOBz as the base is roughly twice as fast as with TBAOH (1.5 × 10^−9^ M s^−1^*vs.* 7.0 ± 0.2 × 10^−10^ M s^−1^). Both reactions involve **R1** as a common pre-equilibrium, and have the same *k*_f,_**R2** and *k*_f,_**R3**_/Bz*OH_ rate constants. The primary difference between the two initial rates is in the different concentrations of Bz*OH. Simulations, including all possible hetero-/homoconjugations, reveal that the total initial concentration of benzoic acids for the TBAOBz case is 4.2 mM *versus* 1.3 mM with TBAOH. This predicts a tripling of the initial H/D exchange rate, which is in reasonable agreement with the observed factor of 2 (note that this analysis ignores any contribution from the *k*_f,_**R3**_/MeOH_ pathway). The reaction that governs the H/D exchange rate in both the TBAOBz and TBAOH cases appears to be exchange between **2C−OD** and Bz*OH (**R3**), as implicated in our previous studies.^8^ The direct protonation of the fluorenyl anion (**R2**) by Bz*OH could be competitive, if there is a slower-than-expected rate of H/D exchange between benzoic acids (**R3**), as discussed above.

The overall success of the simulations to qualitatively capture many aspects of the H/D exchange reactions supports the kinetic model. The fits to the model indicate that the main mechanism for H/D exchange is the **R3** step. Most importantly, the results support the small experimental value of *K*_eq,_**R1**. This value of *K*_eq,_**R1** rules out the proposed PT-first mechanism for the fluorenyl-benzoate oxidation reactions.

### (IV) Asynchronous or imbalanced PCET

The critique by CS was apparently motivated by their belief that CPET cannot occur in an asynchronous, asymmetric, or imbalanced reaction, *i.e.*, weighing the proton and electron transfer components differently. This important and not unreasonable intuition appeared in a different form and context in a recent perspective in *J. Am. Chem. Soc.*^19*c*^ Having demonstrated here that CS's alternative mechanism for our data is incorrect, it is worth dissecting what the experiments tell us and how they might be interpreted theoretically.

The reported kinetic data for the oxidations of substituted fluorenyl benzoates were interesting because the rate constants did not follow simple Marcus-type behaviour, in which *k*_CPET_ should vary only with the overall 
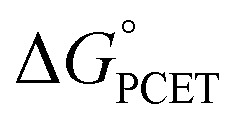
 and not with how that 
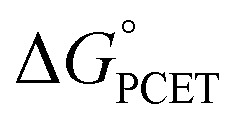
 was obtained.^10^ As noted above, the rate constants were less sensitive to changes in the electron transfer component, with a constant Brønsted *α* of 0.21 ± 0.2 upon varying the outer-sphere oxidant by 1.15 V. The reactions were relatively more sensitive to changes in substituents on the benzoate group (mostly 0.48 < *α* < 0.61), which (we argued) primarily affected the proton transfer coordinate. This represents an unusual experimental imbalance in how the reaction rates respond to different ways that the Δ*G*° is changed across the series. This observation is perhaps related to Srnec's recent proposal that DFT-computed reaction barriers are lower when the “asynchronicity” of a CPET reaction is larger, defining an asynchronicity parameter as the difference between the ET and PT components of the 
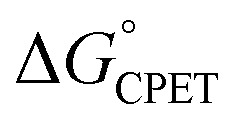
.^29^

From a theoretical perspective, the dominant picture of CPET reactions is based on a Marcus theory framework where both the electron and proton are treated as quantum particles. In this model, neither the electron nor the proton appears in the reaction coordinate (*i.e.*, they are described by a wavefunction, not a defined position). The reaction coordinate involves heavy-atom rearrangements of the reactants and the surrounding solvent molecules. The heavy atoms rearrange to configurations along a multidimensional seam, on which the reactant and product diabatic free energy surfaces are isoergic for CPET and a simultaneous double tunnelling of the e^−^ and H^+^ can occur. In this model, the actual transfer of the electron and proton are instantaneous and cannot be asynchronous, which we believe is the origin of the intuition described above. Still, this model is not the last word in PCET reactions, as shown by very recent studies on ultrafast timescales that show asynchronous proton and electron transfers even for concerted reactions (reactions without an intermediate along the reaction coordinate).^30^

We consider here four of the parameters in the Marcus-type double-tunnelling model. The driving force 
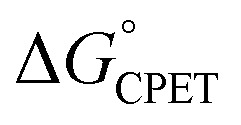
 and the reorganization energy *λ* combine to give the Marcus barrier to the reaction (Δ*G*^‡^ = (Δ*G*° + *λ*)^2^/2*λ* in the weak-coupling limit). This barrier is the free energy at the configuration of the intersection of the Marcus parabolas. The double-tunnelling does not typically occur at this configuration, however, as the tunnelling probabilities (*V*^2^) are larger at configurations with shorter proton donor–acceptor distances *R*. This is because *V*^2^ involves overlaps between reactant and product proton wavefunctions. The compression along *R* has an energetic cost *U*(*R*) with a concomitant decrease in Boltzmann population. The 
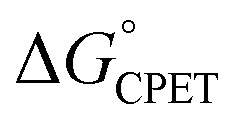
 by definition involves the PT and ET components equally and has no asymmetry (at least when ignoring contributions from proton vibrational excited states). The *U*(*R*) and *V*^2^ terms, however, will likely respond differently to changes in the PT *vs.* the ET components of a CPET reaction. Most notably, PT is much more sensitive to small changes in donor–acceptor distance than ET: modelling the distance dependences as e^−*βr*^, *β*_ET_ ∼ 1 Å^−1^ while *β*_PT_ ∼ 15–30 Å^−1^.^31–33^

Our suggestion of asynchronicity was based on the presence of multiple linear free energy relationships (LFERs) across the series of reactions. Reactions with the same value of 
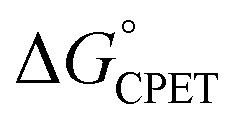
 gave different values of *k*_CPET_ depending on how that 
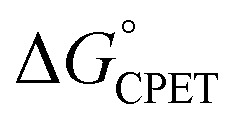
 was obtained. The theoretical examination by Sayfutyarova *et al.* indicated that this was due to slightly different ground state structures of the different compounds, which led to different compression energies *U*(*R*).^17^ More generally, the optimal donor–acceptor distance *R* and the shape of the proton potential energy surface (PES) can be significantly modulated by the p*K*_a_ values of the donor and acceptor, changing both *U*(*R*) and *V*^2^, so that changes in p*K*_a_ can modulate rate constants differently than equivalent changes in *E*°. In addition, LFER treatments of CPET reactions, including eqn (1) and (2) above, typically assume that *λ* is constant across the series. However, as Marcus pointed out in 1969,^34^*λ* can vary across a series and this will shift the value of the Brønsted *α*. Srnec emphasized that values of *λ* across a series need not weigh the contributions from the PT or ET components equally.^29^ Thus, there are a number of ways in which rate constants for a series of CPET reactions could respond differently to changes in the proton transfer portion of the overall 
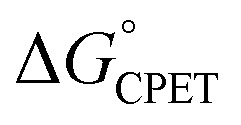
*vs.* the electron transfer portion.

**Fig. 3:**
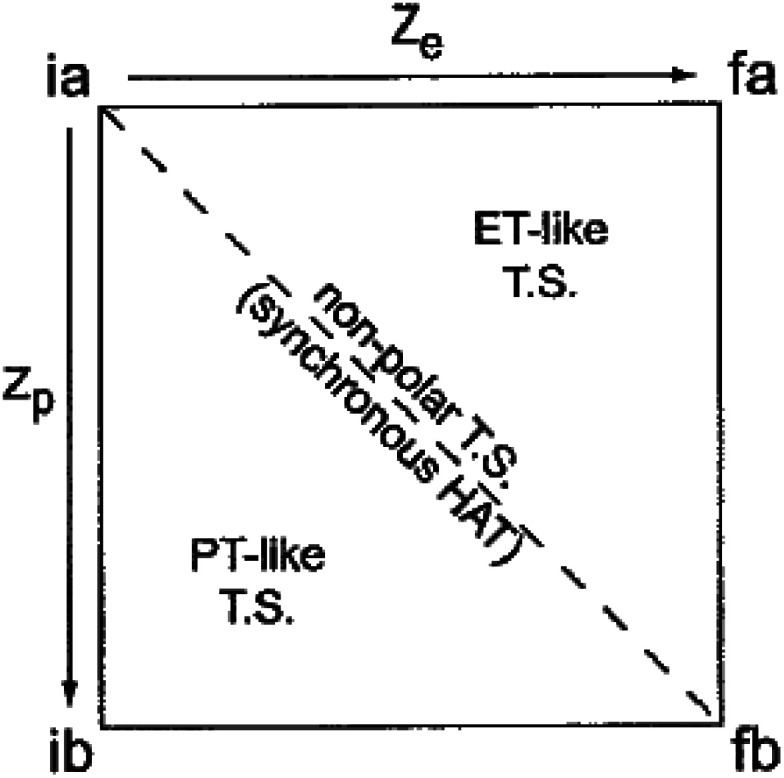
More O'Ferrall–Jencks plot of a PCET reaction and its caption, reprinted from Hodgkiss, Rosenthal and Nocera 2007 with permission.^36^ Copyright 2007 Wiley. “The four-state PCET reaction in a solvent coordinate system. The four states are abbreviated with labels according to the initial and final states of the electron (i and f, respectively) and the initial and final states of the proton (a and b, respectively). The coordinates *z*_e_*vs. z*_p_, refer to the collective solvent coordinates that are coupled to ET and PT, respectively. A concerted PCET reaction can have a trajectory anywhere within this space with a single transition state. The synchronicity of the reaction reflects the nature of this trajectory; the synchronous HAT reaction is defined by the strictly diagonal line, whereas deviation from this line reflects asynchronous PCET with varying degrees of ET or PT character dominating the transition state.”

The experimental results here and elsewhere^11^ that invoke ‘asynchronicity’ involve changes across a group of similar reactions, whereas the term ‘asynchronous’ seems to refer to the properties of a single reaction. The idea that PCET reactions do not have to be symmetrical has a significant history. Hammes-Schiffer's early formulations of multistate continuum theory of PCET invoked different solvent coordinates for these two components, *z*_e_*vs. z*_p_, which extended Marcus 1D parabolas to 2D paraboloids.^35^ Nocera *et al.* in 2007 used these two distinct reaction coordinates for PT and ET to draw a More O'Ferrall–Jencks plot of a PCET reaction and to propose this as a model for asynchronicity (Fig. 3).^36^ More study is needed to determine whether it is common for a CPET reaction to be more advanced along the PT or ET component of the heavy atom reaction coordinate at the configuration that has the maximum contribution to the rate constant. Such an imbalance would be reminiscent of the physical organic principle of non-perfect synchronization, which invokes unbalanced transition states as the origin of the slow deprotonations of many C–H bonds.^37–39^ This concept has been developed in a Marcus framework^34,39,40^ and cited recently to account for two series of H-atom transfer rate constants with unusual rate/driving force relationships.^41^ In light of this prior literature, perhaps ‘non-perfect synchronization,’ or ‘imbalanced’ transfer could be more appropriate terms than ‘asynchronous’. We feel, however, that ‘asymmetric’ PCET is not the best term because readers may confuse that with enantioselective or the like.

## Conclusions

Understanding the origins of rate-driving force relationships is important for the interpretation and application of PCET experiments and theories. Some recent experimental and computational studies of concerted proton–electron transfer (CPET) reactions have shown unusual dependences of the rate constant on driving force, the Brønsted *α*, 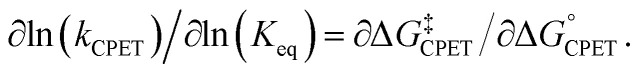 In particular, the fluorenyl-benzoate system that is the focus here showed an unusually small and constant *α* with changes in the oxidant, but larger *α* values for changes in benzoate substituents using the same oxidant. The shallow slope and the lack of a 1 : 1 correspondence of *k*_CPET_ on 
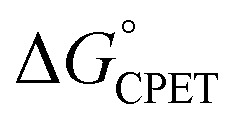
 are outside of a simple Marcus model. In part due to resistance to these conclusions, a recent paper claimed that the reactions did not all proceed by CPET but rather changed from PT-ET to CPET mechanisms over the series, and that this was the origin of the unusual behavior.^13^ The data and analysis in this report rule out this proposal of a mechanism crossover. The initial PT is more uphill than was assumed in ref. 13, and a kinetic analysis of the very slow H/D exchange reactions in the absence of an oxidant is inconsistent with initial PT. CPET reactions should be viewed through the full lens of vibronically nonadiabatic PCET theory, rather than just considering 
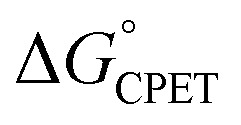
 and the proton/electron tunnelling event that intrinsically deal with the proton and electron components equally. Prior theoretical conclusions indicated that the disparate *α* values derive from the involvement of proton vibrational excited states and changes in ground state structures that reduce the energy to reach configurations with high tunnelling efficiencies. These are changes in the terms for vibronic coupling (*V*^2^) and distortion along the proton donor–acceptor mode (*U*(*R*)) of vibronically nonadiabatic PCET theory that are not constrained to treat changes in PT and ET equally. There are also interesting connections with semiclassical physical-organic proposals of unbalanced transition states, such as the principle of nonperfect synchronization. Thus, sets of CPET reactions can display different sensitivities to changes in the proton and electron components. Additional studies are in progress to explore the prevalence of this behaviour.

## Author contributions

BK performed initial experiments and provided some of the initial structure of the study; SCC performed most of the experiments, worked with ACB on the kinetic modelling, wrote most of the manuscript and brought the project to completion; ACB worked with SCC on the kinetic modelling; JMM oversaw the project, discussed the results, and edited the manuscript.

## Conflicts of interest

There are no conflicts to declare.

## Supplementary Material

SC-012-D1SC03344A-s001
