# Supplementary material for: C–H oxidation in fluorenyl benzoates does not proceed through a stepwise pathway: revisiting asynchronous proton-coupled electron transfer
Source: Chem Sci. 2021 Sep 10;12(39):13127–36. doi: 10.1039/d1sc03344a (PMC8513817; doi:10.1039/d1sc03344a)
Supplement: SC-012-D1SC03344A-s001 [file SC-012-D1SC03344A-s001.pdf]

Electronic Supplementary Material (ESI) for Chemical Science.
This journal is © The Royal Society of Chemistry 2021

C–H Oxidation in Fluorenyl Benzoates Does Not Proceed Through a Stepwise Pathway: Revisiting Asynchronous Proton-Coupled Electron Transfer

Scott C. Coste,[§] Anna C. Brezny,[‡] Brian Koronkiewicz,[§,||] and James M. Mayer*[,§]

[§] Department of Chemistry, Yale University, New Haven, CT 06520-8107, USA

[||] Current address: Research and Exploratory Development Department, The Johns Hopkins University Applied Physics Laboratory, 11091 Johns Hopkins Road, Laurel, Maryland 20723, USA

[‡] Department of Chemistry, Skidmore College, Saratoga Springs, New York 12866, USA

Corresponding author: james.mayer@yale.edu

Revisions in this document are marked with dark red text.

Table of Contents

1 General Considerations 2
2 H/D exchange measurements 2
Experimental details 2
3 pK_a determination of fluorenyl C–H bond in 1 and 2 3
3.1 Experimental details 3
3.2 Figures S1-S3: UV-Vis spectra for titration of **1** with t-BuP_1(pyrr) 4
3.3 Tables showing numerical values from titration of methyl fluorenyl benzoate ester with t-BuP_1(pyrr) 6
3.4 Discussion of potential intramolecular interactions in the fluorenyl benzoate pK_a estimations 6
4 Stopped-flow experiment to estimate $k_{f,R2}$ 8
5 Kinetic modelling for H/D exchange measurements 9
5.1 Discussion on rate constant estimations and fitting 9
5.2 Figure S5 Determination of $k_{f,R3/MeOH}$ 10
5.3 Additional fits using parameters from Costentin *et al*. 11
References 12

1 General Considerations

Sodium methoxide (NaOMe) (Sigma Aldrich, 95%), tetra-n-butylammonium hydroxide (TBAOH) (1.0 M in MeOH, Sigma Aldrich, Lot #STBH8281), tetra-n-butylammonium benzoate (TBAOBz) (Sigma Aldrich, >99%), and were purchased from commercial suppliers and used as received. The actual concentration of the TBAOH solution in MeOH was determined to be 0.979 M by ^{1}H NMR using an internal standard. *tert*-Butylimino-tri(pyrrolidino)phosphorane (*t*-BuP_1(pyrr)) (Sigma Aldrich, ≥97.0%) was vacuum distilled and stored at −35 °C in a N_2-filled glovebox. Acetonitrile (MeCN) was Burdick Jackson low water grade and sparged with Argon. Methanol (MeOH) and isopropanol (IPA) were dried over 4 Å sieves and vacuum distilled. d_3-MeCN was purchased from Cambridge Isotope Laboratories and freeze-pump-thawed and run through dry alumina. Methyl 2-(9H-fluorneyl) benzoate ester[1] (**1**) and 2-(9D-fluorenyl) benzoic acid[2] (**2-*d***) were synthesized as described previously. All manipulations and experiments were done in a dry N_2-filled glovebox or in J. Young tubes.

^{1}H nuclear magnetic resonance (NMR) spectra were collected on an Agilent DD2-400 MHz spectrometer at room temperature and referenced to residual protio solvent peaks. Optical UV-Visible spectra were measured on Agilent Cary 5000 or Cary 60 spectrometers at room temperature in N_2-filled gloveboxes via fiber optics cables.

2 H/D exchange measurements

Experimental details

H/D exchange experiments were done by dissolving 3.0 mg (0.01 mmol **1CD** in 800 μL d_3-MeCN. Then 0.9 eq of base was added (9.6 μL of 0.979 M TBAOH in MeOH or 3.4 mg TBAOBz) followed by addition of 16.2 μL MeOH. The mixture was prepared in the glovebox and added to a J. Young tube. Between ^{1}H NMR measurements, the NMR tube was stored at room temperature.

3 pK_a determination of fluorenyl C–H bond in 1 and 2

3.1 Experimental details

The fluorenyl 9-H C–H bond pK_a was determined spectrophotometrically through titrations with the phosphazene base, t-BuP$_1$(pyrr) (pK_a = 28.42 in MeCN).[3] Stock solutions of the phosphazene base (**B**) and the ester (**C–H**) were freshly prepared in anhydrous MeCN prior to titration measurements.

For the titrations, a known volume of the **C–H** stock solution was added to 2.0 mL MeCN. Then, a known volume of **B** stock solution was added and allowed to equilibrate for 10 minutes, as monitored by the carbanion absorbance. Once equilibrium was reached, an optical spectrum was measured, and this was repeated multiple times. Following the last addition, lutidinium tetrafluoroborate was added to confirm the reversibility of the proton transfer.

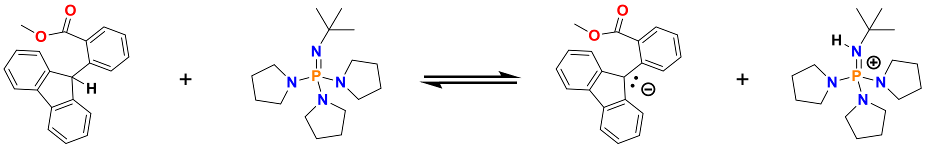

C–H **B** **C** **B –H**

$$K = \frac{[C^-][B^+ - H]}{[C - H][B]}$$

The pK_a of the methyl fluorenyl benzoate ester was determined by plotting the concentrations of [**B**$^+$**–H**] × [**C**$^-$] *vs*. [**C–H**] × [**B**] where the slope of the straight line represents the equilibrium constant, K, of the reaction above. The reported pK_a was calculated by adding the pK_a of t-BuP$_1$(pyrr) to the pK from the titration equilibrium, this was repeated twice. The concentrations of [**C–H**], [**C**$^-$], [**B**], and [**B**$^+$**–H**] at each step were calculated using the absorbance of the carbanion and the moles of corresponding reagents added to the mixture. Uncertainties reported in Tables S1-S2 were estimated based on mass measurement error and standard deviations from absorption spectra. Propagating error to the final value yields a smaller uncertainty than reported here, we report uncertainty in the pK_a values to the tenth place because of slight curvature and the non-zero intercept in the equilibria plots. The molar absorptivity of the ester carbanion was obtained by a separate titration of methyl fluorenyl benzoate ester in MeCN with a sodium methoxide solution in anhydrous, degassed isopropanol (Figure S1); λ_{max}, nm (ε, M^{-1} cm^{-1}): 364 (13,000), 414 (9,400), 480 (8,300), 504 (sh).

3.2 Figures S1-S3: UV-Vis spectra for titration of **1** with t-BuP$_1$(pyrr)

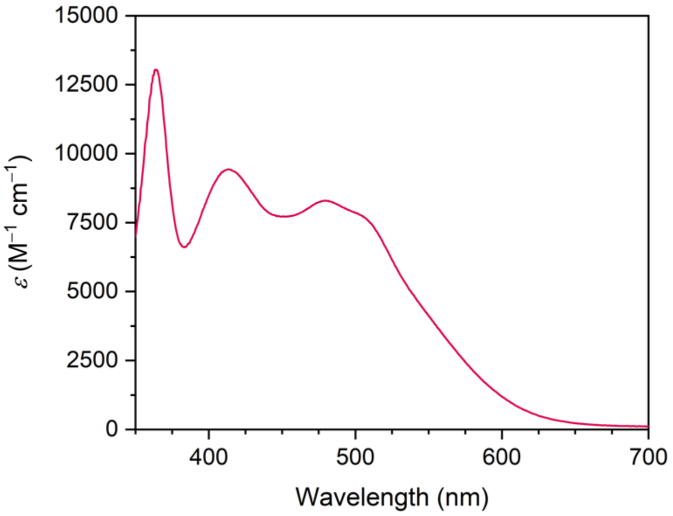

Figure S1. UV-Vis spectrum of 63.1 μM methyl fluorenyl benzoate ester carbanion (**2-OMe,C**$^-$) in 2 mL MeCN at room temperature generated from the addition of 3 molar equivalents of NaOMe in 4 μL of ^{i}PrOH.

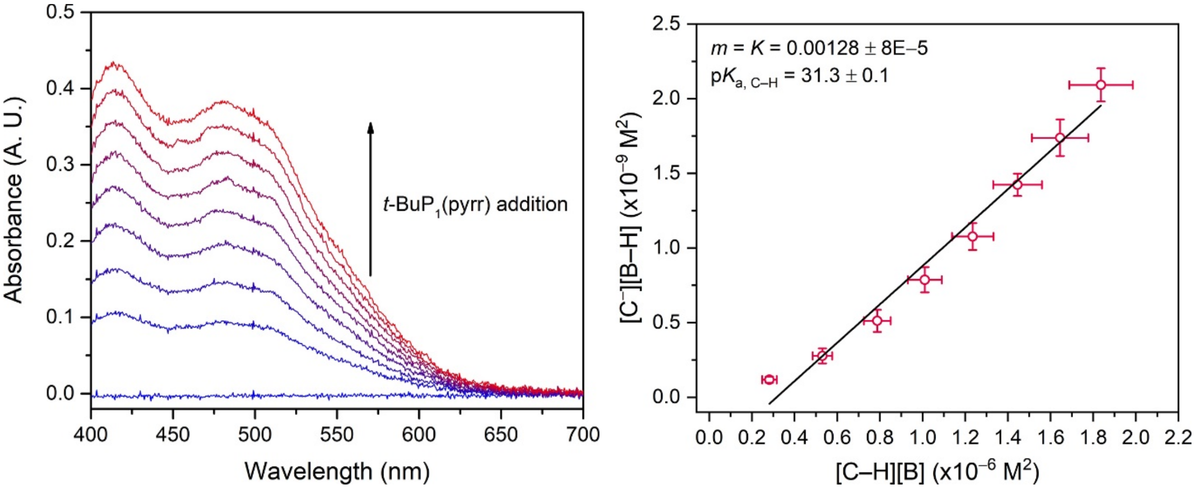

Figure S2. (left) UV-Vis spectra of titrating 1.3 mM methyl fluorenyl benzoate ester with *t*-BuP$_1$(pyrr) in MeCN at room temperature. (right) Plot of [**B**$^+$**–H**] × [**C**$^-$] *vs*. [**C–H**] × [**B**] depicting *K*. The non-zero intercept and curvature is likely the result of a small concentration of acidic impurities in the MeCN solution or because not enough time was allowed for the titration point to equilibrate.

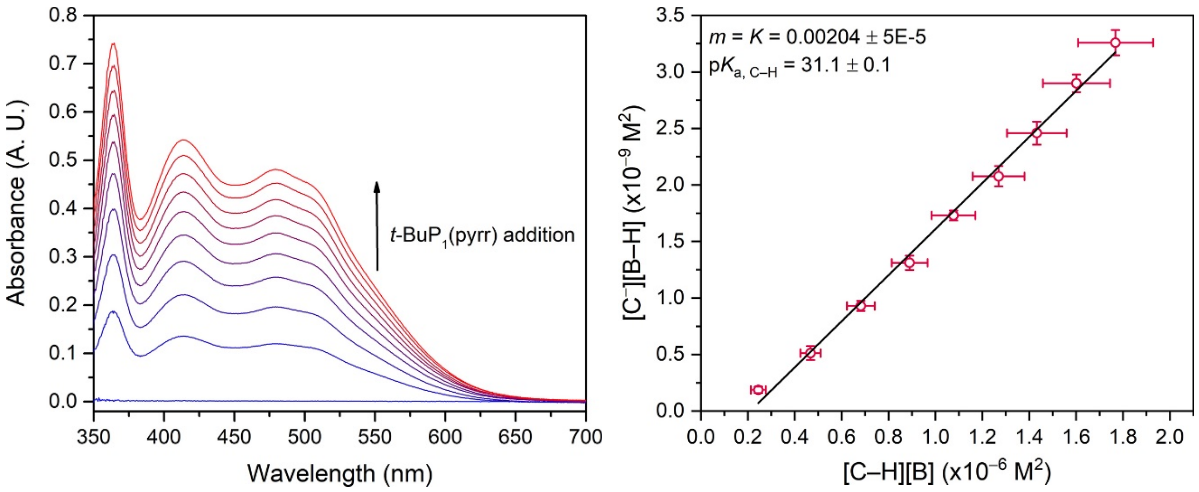

Figure S3. (left) UV-Vis spectra of titrating 1.2 mM methyl fluorenyl benzoate ester with *t*-BuP$_1$(pyrr) in MeCN at room temperature. (right) Plot of [**B$^+$–H**] × [**C$^-$**] *vs*. [**C–H**] × [**B**] depicting *K*. The non-zero intercept and curvature is likely the result of a small concentration of acidic impurities in the MeCN solution or because not enough time was allowed for the titration point to equilibrate.

3.3 Tables S1-S2 numerical values from titration of methyl fluorenyl benzoate ester with t-BuP$_1$(pyrr)

Table S1. Numerical values (and uncertainties) of reagents based on absorbance values and mass balance for each titration step for Figure S2.

Titration step	[**C**$^-$] (M)	[**B**$^+$–**H**] (M)	[**C–H**] (M)	[**B**] (M)
1	1.1±0.1 × 10^{-5}	1.1±0.1 × 10^{-5}	1.3±0.1 × 10^{-3}	2.2±0.2 × 10^{-4}
2	1.7±0.2 × 10^{-5}	1.7±0.2 × 10^{-5}	1.24±0.9 × 10^{-3}	4.3±0.2 × 10^{-4}
3	2.3±0.2 × 10^{-5}	2.3±0.2 × 10^{-5}	1.23±0.9 × 10^{-3}	6.4±0.2 × 10^{-4}
4	2.8±0.2 × 10^{-5}	2.8±0.2 × 10^{-5}	1.20±0.9 × 10^{-3}	8.4±0.2 × 10^{-4}
5	3.3±0.2 × 10^{-5}	3.3±0.2 × 10^{-5}	1.18±0.9 × 10^{-3}	1.05±0.02 × 10^{-3}
6	3.7±0.1 × 10^{-5}	3.7±0.1 × 10^{-5}	1.16±0.9 × 10^{-3}	1.24±0.02 × 10^{-3}
7	4.2±0.2 × 10^{-5}	4.2±0.2 × 10^{-5}	1.14±0.9 × 10^{-3}	1.44±0.02 × 10^{-3}
8	4.6±0.2 × 10^{-5}	4.6±0.2 × 10^{-5}	1.12±0.9 × 10^{-3}	1.63±0.02 × 10^{-3}

Table S2. Numerical values (and uncertainties) of reagents based on absorbance values and mass balance for each titration step for Figure S3.

Titration step	[**C**$^-$] (M)	[**B**$^+$–**H**] (M)	[**C–H**] (M)	[**B**] (M)
1	1.4±0.2 × 10^{-5}	1.4±0.2 × 10^{-5}	1.2±0.1 × 10^{-3}	2.1±0.2 × 10^{-4}
2	2.3±0.2 × 10^{-5}	2.3±0.2 × 10^{-5}	1.17±0.9 × 10^{-3}	4.1±0.2 × 10^{-4}
3	3.1±0.1 × 10^{-5}	3.1±0.1 × 10^{-5}	1.15±0.9 × 10^{-3}	6.1±0.2 × 10^{-4}
4	3.6±0.1 × 10^{-5}	3.6±0.1 × 10^{-5}	1.13±0.9 × 10^{-3}	8.1±0.2 × 10^{-4}
5	4.2±0.1 × 10^{-5}	4.2±0.1 × 10^{-5}	1.11±0.9 × 10^{-3}	1.01±0.02 × 10^{-3}
6	4.6±0.1 × 10^{-5}	4.6±0.1 × 10^{-5}	1.10±0.9 × 10^{-3}	1.20±0.02 × 10^{-3}
7	5.0±0.1 × 10^{-5}	5.0±0.1 × 10^{-5}	1.08±0.9 × 10^{-3}	1.39±0.02 × 10^{-3}
8	5.4±0.1 × 10^{-5}	5.4±0.1 × 10^{-5}	1.07±0.9 × 10^{-3}	1.58±0.02 × 10^{-3}
9	5.7±0.1 × 10^{-5}	5.7±0.1 × 10^{-5}	1.06±0.9 × 10^{-3}	1.76±0.02 × 10^{-3}

3.4 Discussion of potential intramolecular interactions in the fluorenyl benzoate pK_a estimations

We note that DFT calculations from our original report of this system[2] indicated that the closed conformer of **2C⁻** (where the carboxylic acid proton faces towards the carbanion, C⁻···H–O) is stabilized by 7.7 kcal mol^{-1} relative to the open conformer (where the carboxylic acid proton faces away from the carbanion). This would suggest a stabilizing non-classical C⁻···H–O interaction that would not be present in the ester derivative used to estimate $K_{eq,R1}$, and therefore a systematic error in the analysis. A prior paper reported the existence of similar C⁻···H–O interactions in *alcohol*-fluorenide anions, which is qualitatively consistent with the computed stabilization of the closed conformer in **2C⁻**.[4] In some molecules, this interaction is strong enough to compete with classical hydrogen bonding to the DMSO solvent.

The goal of our prior DFT calculation was to demonstrate that a non-classical hydrogen bond was occurring between the carbanion and the benzoic acid. This calculation was not designed to provide quantitative information about the value of $K_{eq,R1}$ (as stated explicitly in the SI).[2] Therefore, the DFT calculations in ref 2 were done without any explicit solvent, only with a polarizable continuum solvation model equivalent to the dielectric of acetonitrile. So, this does not include the stabilization from the *classical* hydrogen bonding with solvent or methanol present, which is expected to have similar strength.[4] When these two are of equal strength, the open and closed conformers will be present in roughly equal concentrations and there is only a small additional stabilization of the C⁻···H–O form (~ -0.4 kcal mol^{-1} = $RT\ln(2)$). The open conformer with the classical hydrogen bond (O–H···NCMe or O–H···OHMe) should be present at equilibrium in these slow reactions and would be the conduit for the H/D exchange reaction. Additionally, the non-classical C⁻···H–O interaction in 2C⁻ should be about as strong as hydrogen bonding to the solvent in compound 1, implying there is little change in the hydrogen bonding on conversion of **1** to **2C⁻**. Therefore, using the methyl ester is a good model for indirectly measuring $K_{eq,R1}$ (see top of Scheme 2) because the solvation of the ester should also not significantly change on converting **1OMe** to **2C⁻OMe**.

4 Stopped-flow experiment to estimate $k_{f,R2}$

The kinetics of protonating the methyl ester fluorenide **2C⁻OMe** with benzoic acid was monitored by the disappearance of the red-colored carbanion in the visible spectrum in a rapid-mixing stopped-flow instrument. Solutions of **2C⁻OMe** (0.2 mM) and benzoic acid (0.2 mM) in MeCN were prepared in an N_2-filled glovebox, and then loaded into 5 mL gas-tight syringes (Restek). The stopped-flow instrument was flushed with 20 mL dry, degassed MeCN prior to measurements. Reagent concentrations are halved in the stopped-flow mixing chamber.

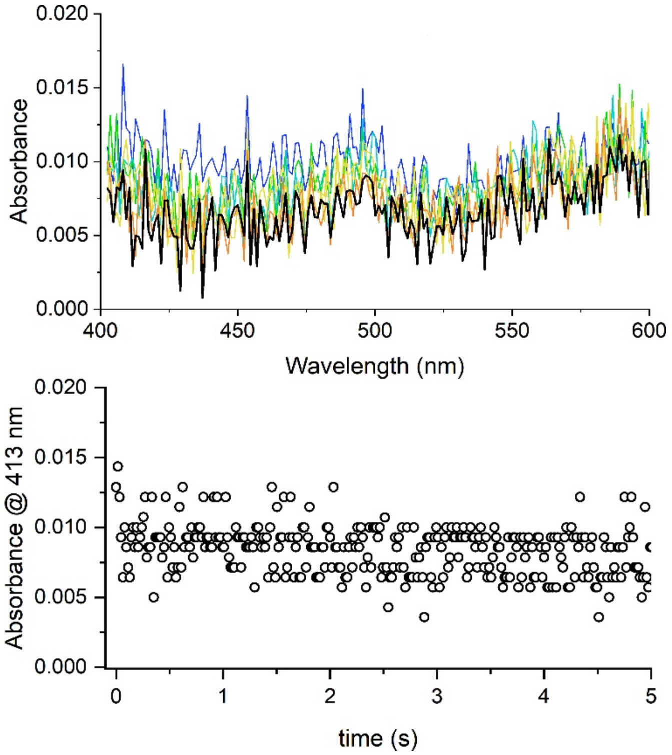

Figure S4. Stopped-flow kinetics data for the reaction of benzoic acid with methyl ester fluorenide **2C⁻OMe** in MeCN at room temperature. The red carbanion is almost entirely consumed prior to the first data point after the mixing time (~5 ms). This provides a rough estimate of $k_{f,R2}$ to be about 1×10^6 M^{-1} s^{-1}.

5 Kinetic modelling for H/D exchange measurements

5.1 Discussion on rate constant estimations and fitting

The rate and equilibrium constants used in the COPASI fitting are given in Table S1 below. We assume that proton exchange between benzoic acid groups ($k_{\mathrm{f,R3/BzOH}}$) occurs with a rate constant at the diffusion limit, $10^{10.2}$ $\mathrm{M^{-1}\ s^{-1}}$,[5] by diffusion limited formation of the hydrogen bonded dimer followed by rapid double-proton exchange.[6] The main text discusses the changes that occur in the model if a smaller value of $k_{\mathrm{f,R3/Bz^*OH}}$ is used. All hetero-/homoconjugation forming reactions were assumed to proceed at the diffusion limit and with equilibrium constants of 10^3 $\mathrm{M^{-1}}$.[7] To model H/D exchange between the benzoic acid group in **2** and MeOH (**R3**), the rate of proton exchange between MeOH and benzoic acid ($k_{\mathrm{f,R3/MeOH}}$) was measured by lineshape analysis of the ^{1}H NMR spectrum in d_3-MeCN (details below). Fluorenyl anion protonation by MeOH was omitted from the model owing to its low rates (in DMSO) compared to protonation by exogenous Bz*OH.[8] The parameters $K_{\mathrm{eq,R1}}$ and $k_{\mathrm{f,R2}}$ were fit to the H/D exchange data; $k_{\mathrm{r,R2}}$ was set to equal $k_{\mathrm{f,R2}}/10^{9.9}$ based on our pK_a measurements in Section I of the main text. The correlation matrices indicated that both fitted parameters were not dependent on each other as an indication the model was not overparameterized.

To estimate $k_{\mathrm{f,R3/MeOH}}$, we measured the methanolic O–H proton linewidth ($\nu_{1/2}$) in 0.5 MeOH solutions (in d_3-MeCN) with and without 0.01 M BzOH present (Figure S6). The linewidth of the exchanging methanolic proton, in the presence of BzOH, was converted to an exchange rate (k_{ex}) using the following equation at the intermediate exchange limit[9]:

$$k_{\mathrm{ex}} = \pi \nu_{1/2} - 1/T^*_2$$

where T^*_2 is the effective T_2 measured by the methanolic O–H proton linewidth in the absence of BzOH. This was then converted to a bimolecular proton exchange rate constant ($k_{\mathrm{f,R3/MeOH}}$) by the following relation[10]:

$$k_{\mathrm{ex}} = k_{\mathrm{f,R3/MeOH}}[\mathrm{Bz^*OH}]$$

To include this process in the COPASI kinetic model, we assumed the bimolecular rate law below. While this reaction may be acid or base catalyzed, the value of $k_{\mathrm{f,R3/MeOH}}$ can be used directly in our COPASI model because it was determined under similar concentrations for H/D exchange.

$$\mathrm{rate} = k_{\mathrm{f,R3/MeOH}}[\mathrm{Bz^*OH}][\mathrm{MeOH}]$$

Correlation (covariance) matrix for fit shown in Figure 2 of main text:

$$\begin{array}{c} \\ K_{eq,R1} \\ k_{f,R2} \end{array} \begin{array}{c} \begin{array}{cc} K_{eq,R1} & k_{f,R2} \end{array} \\ \begin{bmatrix} 1 & -0.775 \\ -0.775 & 1 \end{bmatrix} \end{array}$$

5.2 Figure S5 Determination of $k_{f,R3/MeOH}$

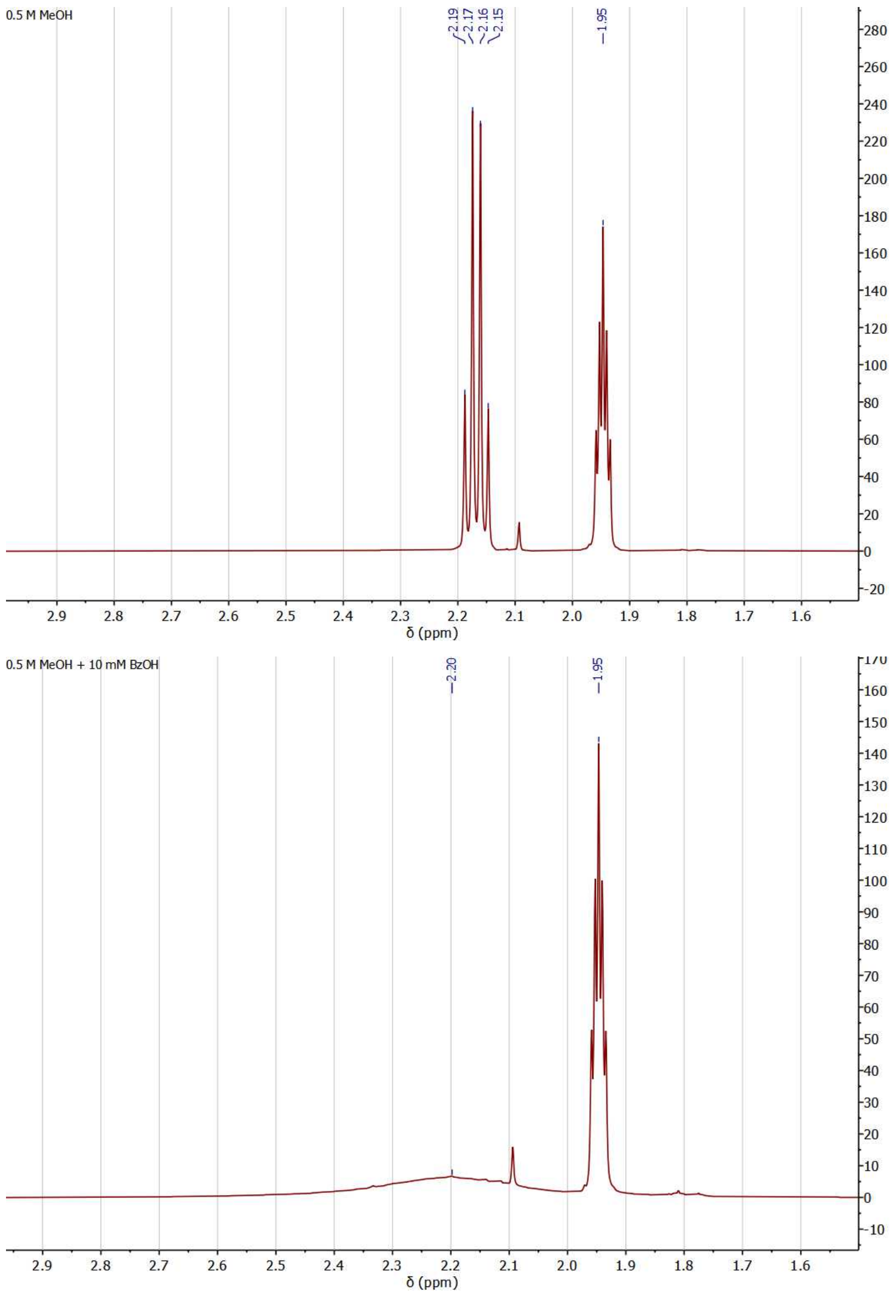

Figure S5. ^{1}H NMR spectra of 0.5 M MeOH in d_3-MeCN (top) and 0.5 M MeOH + 0.01 M BzOH in d_3-MeCN (bottom) used for linewidth analysis. Both spectra were measured in dry d_3-MeCN at room temperature using the same spectral acquisition parameters. The peak at δ~2.09 ppm is from an acetone impurity.

5.3 Additional fits using parameters from Costentin et al.

Table S3. Fit using the kinetic model when $k_{f,R3/Bz*OH}$ is set to 0 M^{-1} s^{-1} using $K_{eq,R1}$, $k_{f,R1}$, and $k_{r,R1}$ from Costentin *et al*. This shows the reaction is still too fast even when the main exchange mechanism is that considered by Costentin *et al*.

Parameter	Value in Model
$K_{eq,R1}$	1.5×10^{-5} [a]
$k_{f,R1}$	4.3×10^{4} s^{-1} [a]
$k_{r,R1}$	2.9×10^{9} s^{-1} [a]
$k_{f,R2}$	$5.3 \pm 600 \times 10^{6}$ M^{-1} s^{-1} [b]
$k_{r,R2}$	$6.6 \pm 600 \times 10^{-4}$ M^{-1} s^{-1} [c]
$k_{f,R3/MeOH}$	3.2×10^{4} M^{-1} s^{-1} [d]
$k_{f,R3/Bz*OH}$	0 M^{-1} s^{-1}

[a] Parameters set from Costentin et al.[11] [b] Parameters fit using COPASI program.
[c] $k_{r,R2}$ set equal to $k_{f,R2}/10^{9.9}$. [d] Obtained from linewidth measurements.

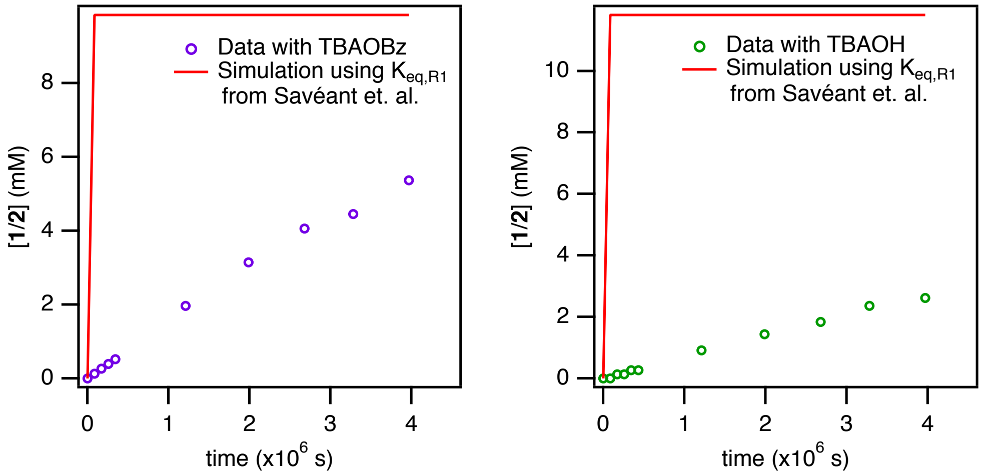

Figure S6. Concentration of protio 2-fluorenyl benzoate (1/2) over time at room temperature with TBAOBz (left) or TBAOH (right) as a base (open circles) as measured by integration of ^{1}H NMR spectra. The solid red lines are the predicted H/D exchange using parameters from the fits presented in Table S1.

References

(1) J.-J. Chen, S. Onogi, Y.-C. Hsieh, C.-C. Hsiao, S. Higashibayashi, H. Sakurai and Y.-T. Wu, *Adv. Synth. Catal.*, 2012, **354**, 1551-1558.

(2) T. F. Markle, J. W. Darcy and J. M. Mayer, *Sci. Adv.*, 2018, **4**, eaat5776.

(3) I. Kaljurand, A. Kütt, L. Sooväli, T. Rodima, V. Mäemets, I. Leito and I. A. Koppel, *J. Org. Chem.*, 2005, **70**, 1019-1028.

(4) I. McEwen, M. Roennqvist and P. Ahlberg, *J. Am. Chem. Soc.*, 1993, **115**, 3989-3996.

(5) J. N. Schrauben, M. Cattaneo, T. C. Day, A. L. Tenderholt and J. M. Mayer, *J. Am. Chem. Soc.*, 2012, **134**, 16635-16645.

(6) (a) Z. Smedarchina, A. Fernández-Ramos and W. Siebrand, *J. Chem. Phys.*, 2005, **122**, 134309. (b) Y. Masuda, T. Nakano and M. Sugiyama, *J. Phys. Chem. A*, 2012, **116**, 4485-4494.

(7) I. M. Kolthoff and M. K. Chantooni Jr., *J. Phys. Chem.*, 1966, **70**, 856-866.

(8) C. D. Ritchie and R. E. Uschold, *J. Am. Chem. Soc.*, 1968, **90**, 3415-3418.

(9) J. Sandström, *Dynamic NMR Spectroscopy*, Academic Press, 1982.

(10) C. Maclean and E. L. Mackor, *Discuss. Faraday Soc.*, 1962, **34**, 165-176.

(11) C. Costentin and J.-M. Savéant, *Chem. Sci.*, 2020, **11**, 1006-1010.
